# Addressing complexity when developing an education program for the implementation of a stroke Electronic Medical Record (EMR) enhancement

**DOI:** 10.1186/s12913-023-10314-z

**Published:** 2023-11-24

**Authors:** Samantha T. Robertson, Ingrid C. M. Rosbergen, Sandra G. Brauer, Rohan S. Grimley, Andrew Burton-Jones

**Affiliations:** 1 School of Health and Rehabilitation Sciences, The University of Queensland, Brisbane, QLD Australia; 2 Sunshine Coast University Hospital, Sunshine Coast Hospital and Health Service, Birtinya, QLD Australia; 3 Digital Health CRC, Sydney, NSW Australia; 4 Department of Physical Therapy & Faculty of Health, University of Applied Sciences Leiden, Leiden, The Netherlands; 5 School of Medicine and Dentistry, Griffith University, Birtinya, Australia; 6 School of Business, University of Queensland, Brisbane, Australia

**Keywords:** Digital intervention, Hospital, Education and training, Implementation science, NASSS, Digital health

## Abstract

**Background:**

Digital interventions in health services often fail due to an underappreciation of the complexity of the implementation. This study develops an approach to address complexity through an evidenced-based, theory-driven education and implementation program for an Electronic Medical Record (EMR) digital enhancement for acute stroke care.

**Methods:**

An action research approach was used to design, develop, and execute the education and implementation program over several phases, with iterative changes over time. The study involved collaboration with multiple statewide and local key stakeholders and was conducted across two tertiary teaching hospitals and a regional hospital in Australia.

**Results:**

Insights were gained over five phases. Phase 1 involved a review of evidence that supported blended learning strategies for the education and training of staff end-users. In Phase 2, contextual assessment was conducted via observation of study sites, providing awareness of local context variability and insight into key implementation considerations. The Non-adoption, Abandonment, Scale-Up, Spread and Sustainability (NASSS) framework assisted in Phase 3 to identify and manage the key domains of complexity. Phase 4 involved the design of the program which included group-based training and an e-learning package, endorsed and evaluated by key leaders. Throughout implementation in Phase 5, further barriers were identified, and iterative changes were tailored to each context.

**Conclusions:**

The NASSS framework, combined with a multi-phased approach employing blended learning techniques, context evaluations, and iterative modifications, can serve as a model for generating theory-driven and evidence-based education strategies that adresss the complexity of the implementation process and context.

**Supplementary Information:**

The online version contains supplementary material available at 10.1186/s12913-023-10314-z.

## Background

Healthcare organizations such as hospitals are amongst the most complex environments for intervention implementation [1]. Implementation of *digital* interventions is especially challenging and failures are frequent [2]. Many elements can impact the success or failure of digital interventions, including context specific factors, healthcare provider attitudes, skills and behaviors, technical capabilities, and policy frameworks [3–7].

Assessing context during the implementation of healthcare interventions is critical [8–10]; however, context is still poorly understood and defined in implementation research [11, 12]. Context can refer to the physical environment in which practice takes place such as the setting or geographical characteristics [13, 14], or more dynamic, social and organizational factors such as individual perceptions, organizational culture, leadership or political influences [11, 15]. Researchers have called for greater clarity in defining context to assist in its appropriate assessment, acknowledging context is not just “a backdrop for implementation” [9] but rather interacts with, and is influenced by the intervention [8, 10, 16]. Particularly overlooked are the evaluation and consideration of team contextual elements. When conducting interventions in team settings, Rogers et al. [11] advises taking into account the contextual variables of the individual, team, organization, and external environment [11].

As implementation in complex environments remains a challenge, complexity science provides a valuable lens through which to view the implementation process [17]. The complexity science approach recognises how individuals and systems are connected and interdependent, and how these relationships lead to changing behaviors [16]. The hospital setting can be described as a Complex Adaptive System (CAS) in which multiple people, processes and practices converge [9]. A CAS can be defined as a “dynamic, self-similar collectivity of interacting, adaptive agents and their artefacts” [16]. That is, a CAS has the capability to “self-organize, accommodate to behaviors and events, learn from experience, and dynamically evolve” [16, 18], but not necessarily in ways we can forecast with confidence. Acknowledging complexity within implementation will allow more agile and non-linear approaches to enhance adoption and uptake of digital health interventions.

Education and training play a crucial role in the implementation of digital interventions in hospitals by equipping healthcare professionals with knowledge and skills, enabling them to adapt to change, and enhancing the uptake of new technologies [19–22]. The importance of education and training in implementation efforts is emphasised by numerous implementation frameworks. For instance, the Consolidated Framework for Implementation Research (CFIR) identifies ‘access to knowledge and information’ as a key construct within the implementation or delivery of an intervention [23]. The Technology Acceptance Model (TAM) [24] also recognizes that training and education can influence the perceived ease of use and usefulness of technology [25], thus affecting its successful implementation. Despite acknowledging education as a key component of the implementation process, few studies provide pragmatic approaches to developing such education programs for implementation [26, 27]. Researchers have stressed that evidenced-based, theory-driven approaches to underpin education programs are needed [4, 28–31], including their detailed descriptions, which are currently lacking [27, 32]. To advance implementation science and its approaches, clear reporting of the strategies behind the development of education and implementation programs is required [32].

This study responds to this need by using the Non-Adoption, Abandonment, Scale-Up, Spread and Sustainability (NASSS) framework [33] and the associated Complexity Assessment Toolkit (NASSS-CAT) [34] to identify complexity and contextual influences in implementation of an Electronic Medical Record (EMR) enhancement for stroke management. The results are used to tailor education and training, which includes addressing challenges within the domains identified as the most complex. The NASSS framework was designed for implementation of technology-based interventions and encompasses not only factors related to implementation but those that influence the adoption, abandonment, and scalability of digital health technologies [33]. A multi-phased approach was used to combine the NASSS framework with evidence-based education and training strategies for implementation. The NASSS framework has not yet been used to guide the development of an education and implementation program in digital health.

## Methods

### Aim and objectives

This research aimed to develop an evidenced-based, theory-driven education and implementation program to promote uptake of a complex EMR digital enhancement in the acute stroke clinical setting. The first objective was to review the existing best practices in EMR education and training to identify and leverage proven methods and strategies. Understanding that each hospital site may have unique requirements and challenges, our second objective was to identify contextual factors that may influence the education and implementation program. Lastly, we sought to determine the usefulness of employing the NASSS framework to prospectively develop the education and implementation program and address complexity within implementation.

### Study design

This research was conducted as part of a larger pre-post study to assess uptake, use and perceptions of an EMR digital enhancement in the acute stroke hospital setting in Australia. Ethical approvals were granted from the Metro South Human Research and Ethics Committee (HREC/2021/71834) and individual health services.

**Fig. 1:**
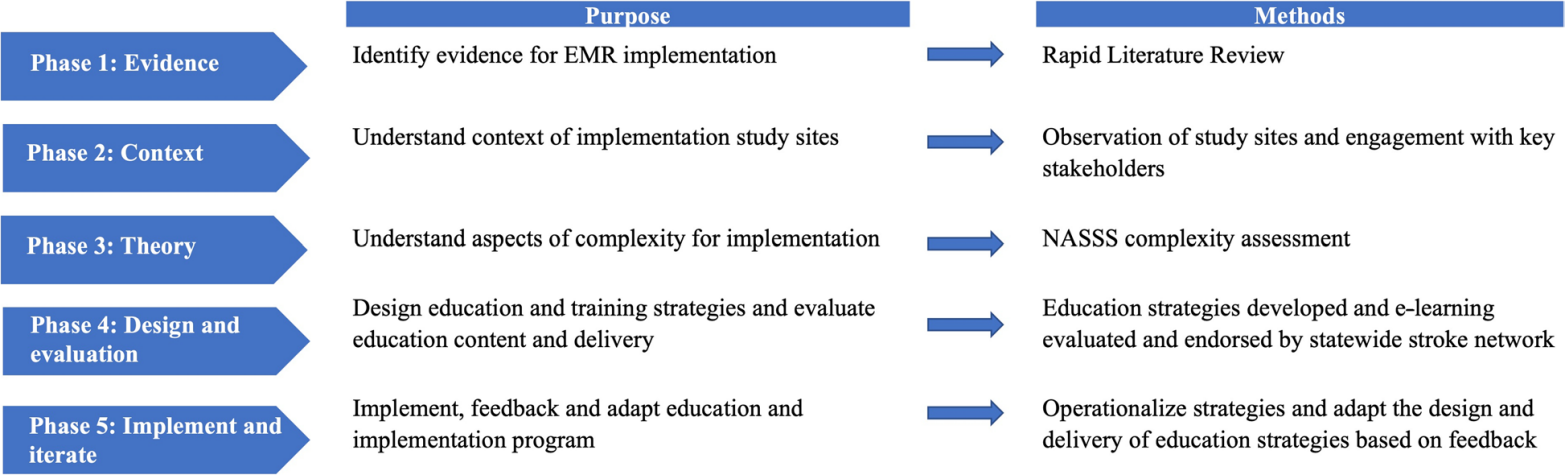
Approach to development of the education and implementation package

The education and implementation program was developed and adapted using an action research approach [35, 36] via a number of phases to gather information from each participating hospital and iteratively re-define how the education and training was delivered. This approach aligns with the required need to account for contextual factors [10, 13, 15] and adaptations over time (Fig. 1).

The research team included academic experts involved in education delivery, implementation and change management, as well as clinical experts in the area of acute stroke care.

### Setting

The study was conducted across three public hospital and health services in Queensland, Australia. Study sites were chosen based on their use of the statewide EMR and included both metropolitan and regional sites:

- Site 1: A teaching hospital with a 707-bed capacity
- Site 2: Large metropolitan teaching hospital with a 906-bed capacity
- Site 3: Regional hospital with a 318-bed capacity

Implementation was targeted at hospital clinical staff (medical, nursing and allied health) working within acute stroke units at participating hospital sites.

### The problem and the digital intervention

The EMR used across the three public hospitals is a Cerner product implemented in 2017 [37]. All health service staff received basic training on its functionality and use. Clinicians within stroke services had reported problems with the EMR due to inefficient documentation, information overload, and inability to automatically extract data for quality improvement [38]. There was a need to modify the EMR to provide more context specific functionality for stroke care which led to development of the stroke EMR enhancement. Stroke clinicians (via a statewide stroke network) and the statewide IT team collaborated to develop a stroke EMR enhancement which was clinician-led in its design. The stroke EMR enhancement sought to offer: 1) better visualization of data, 2) standardized documentation and data collection, and 3) efficient data extraction and use. The enhancement involved creating a single landing page (mPage) offering a consolidated view of a patient’s key clinical information, to enhance information visibility and interprofessional collaboration. Other elements included standardized documentation templates (powerforms) and a data extraction tool for ease of extracting clinical indicator data for use within a national stroke quality registry (see Additional File 1). The enhancement involved numerous design, performance testing and validation phases prior to implementation. The intervention was developed for use in public hospitals across Queensland, Australia.

### Phase 1: evidence

To determine the core components of education and training related to implementation of EMRs or EMR enhancements, PubMed and Scopus were searched for three key concepts: ‘Education and Training’, ‘Electronic Medical Records’ and ‘Staff/Clinicians’. The literature search was restricted to publications from the last 15 years (i.e., from 2007) and employed a snowballing technique via reference list searching and Google Scholar searches. Key citations were used to search the Connected Papers website to identify further publications. The focus of the literature evaluation was on EMR training for health professionals, while excluding EMR training evaluations designed specifically for students or in the undergraduate setting.

### Phase 2: context

Researcher (SR) was directly involved in stroke clinical network meetings about the stroke EMR enhancement in order to understand its background, context, purpose and the design. Furthermore, four key members of the stroke team (medical, nursing, allied health) at the three participating hospital sites were ‘shadowed’ [39, 40] to observe staff interactions with the EMR and with each other, providing contextual insights of each acute stroke ward. Observational shadowing was focused on the *individual* adopter, *team* where the stroke EMR enhancement was implemented and the *organization* involved, including both the statewide organization and the multiple hospital sites adopting the stroke EMR enhancement [11]. Information about existing education and training strategies, staff availability, team meetings, and staff champions was gathered through observational shadowing and direct engagement of each hospital site's learning and development team and ward-based clinical educators.

### Phase 3: theory

To design optimal education and implementation strategies, it is imperative to understand complexity and context. The NASSS theoretical framework addresses multiple levels of complexity, such as the health system, organization, technology, and patient levels [33] and incorporates 7 key domains: 1) condition or illness, 2) technology, 3) value proposition, 4) adopter system, 5) organization, 6) wider system, and 7) process of embedding and adapting over time [33]. It suggests that a technology-supported intervention will be more readily adopted, spread and sustained if all domains are ‘simple.’ In cases where multiple domains are 'complicated', sustaining implementation may prove challenging, costly, and time-consuming, although attainable. If several domains are ‘complex’, this will present a challenge in achieving sustained and widespread adoption of the intervention [41].

The NASSS-CAT was used to identify barriers and understand complexity in the implementation of the stroke EMR enhancement (see Additional File 2) [34]. The evaluation of each complexity domain was conducted by a single researcher (SR), who utilized knowledge sources from statewide meetings regarding the intervention, written audits and reports concerning the technology, key stakeholder engagement and shadowing data. Following identification of the complex domains, the research team discussed education and implementation strategies based on the Effective Practice and Organization of Care (EPOC) taxonomy [42] to mitigate complexity.

### Phase 4: design and evaluation

The research team co-produced an education and training program that incorporated theory, evidence, and contextual factors, and presented this to key stakeholders. Stakeholders were involved in the design and evaluation through informal feedback and a questionnaire, oriented towards identifying factors that may impact uptake at each study site. Key stakeholders were representative of both the broad context (both clinical, represented by the statewide stroke network and technical, represented by the statewide Information Technology (IT) support) and local context (via staff champions, nurse educators, Clinical Nurse Consultants (CNCs) and digital health teams).

Members of the statewide stroke network provided feedback on the education package content via a 7-item questionnaire (5-point Likert scale, strongly disagree to strongly agree). Purposive sampling was used to select participants that were members of the network and had been involved in the design of the stroke EMR intervention.

The CNC from each study site was approached to act as a staff champion for the education, training and implementation of the stroke EMR enhancement. The CNC is an important ‘stroke coordinator’ role described as “facilitating the patient journey through the continuum of care, from the emergency department to discharge, including organizing outpatient follow-up appointments” [43]. CNC responsibilities include data collection, research, education, and service development and improvement [43]. The researcher collaborated with ward CNCs to address practical issues such as how to best involve clinicians in the education.

### Phase 5: implement and iterate

The EPOC taxonomy of implementation strategies for healthcare workers was used to name and define the strategies for education and implementation [42]. Strategies were focused on the NASSS domains of complexity for the stroke EMR enhancement and important contextual considerations for each hospital site. The education and training rollout involved disseminating materials through hospital Learning Management Systems (LMS), with feedback from stakeholders informing changes.

## Results

### Phase 1: evidence for approach, strategies and modality of education and training

The literature review identified three core concepts: 1) approach to training, 2) training strategies and 3) modality of training.

#### Approach to training

There are numerous types of approaches to education and training of staff in using the EMR, such as one-on-one training, peer coaching, computer based training, classroom training and blended methods [44]. Beneficial and negative characteristics of each style of training have been identified, therefore, blended training is recommended to mitigate weaknesses in each individual approach. Blended training is one of the most cited training approaches in the literature [7, 44–47].

#### Training strategies

Key strategies that have been well documented and recommended include assessing baseline computer skills of users [44], scheduling education sessions close to the actual use of the system [48], engaging key stakeholders and staff champions [49–51], providing an incremental approach to training [46] and keeping in mind the social nature of learning [44]. A study by McAlearney et al. [52] describes five propositions that contribute to better learning: (1) emphasizing the positive impact of an EMR, (2) training that contains observation and hands-on activities, (3) clinical champions and positive role-models, (4) building on past computer experiences, and (5) social and cultural sensitivity.

#### Modalities for training

Successful training modalities revolve around maximizing the transfer of learning, as described by Jeyakumar et al. [44] as ‘practice and problem-based learning, integration of learning into practice and enhancing practice improvement and performance.’ Practice and problem-based learning refers to modalities of training such as case-based scenarios and simulation [53, 54] and giving users ‘hands-on’ practice with the system, not just lectures. Team training has been described as covering ‘the big picture’ and allowing teams to recognize each other’s roles and establishing collaborative processes [55]. Team members would visualize each other’s workflows and understand how they interact during patient care.

As a result of the literature review, the following key elements were incorporated into the education and implementation program:

- Blended learning models [44–48, 50, 56–64]
- Engaging key stakeholders and staff champions [44, 49, 50, 52, 65, 66]
- Social nature of learning [44, 52]
- Staged approach to training [46, 67]
- Variety of training modalities e.g. hands on practice [45, 46, 50, 60, 63, 65, 68], video tutorials [69, 70], team training [45, 55, 67, 71]

A particular focus was placed on education and implementation strategies that encourage active participant engagement. Experiential learning theory, which centers learning around experience, inquiry and reflection [72] guided the selection of training modalities.

### Phase 2: context

Twelve staff members were shadowed across the three hospital sites for 40 hours. While a common approach to stroke care was adopted across the hospital sites, differences were observed in the clinical environment (ward communal spaces for staff meetings or interactions, co-location of computer spaces on the ward, availability of resources such as computer workstation on wheels (WOWs) and physical space), and the workforce (clinical staff resources, integration of teams, staff turnover). Differences in team stability were also noted, with Site 1 having a fairly stable and consistent multidisciplinary team (MDT), but high turnover of casual nursing staff, whereas Sites 2 & 3 had a high frequency of staff rotations (especially allied health staff). This presented a challenge in identifying staff to disseminate the education and training, and the requirements of re-training for sustainability of the intervention.

At all hospital sites, clinical ward-based nurse educators played a key role in coordinating training schedules, ensuring staff availability and overseeing general nursing education. This nursing education involved face-to-face sessions prior to the commencement of a shift. Education and training for non-digital interventions primarily involved face-to-face sessions, ward-based MDT education delivered in a lecture-style format, and occasional reference to online tutorials when relevant. Nursing education typically followed a structured, in-person format, while allied health staff engaged in education activities intermittently, often depending on their individual schedules. In contrast, medical staff rarely played an active role in MDT education sessions unless they were the educators themselves.

Upon commencement of employment, all staff received hands-on computer-based training on general use of the EMR, however no re-training schedules were offered. Informal opportunities for EMR education and training were observed within and between professions. Staff members often learned from their peers through impromptu demonstrations, or across professions during MDT meetings or team scrums. New digital updates or changes to the EMR were communicated to staff through email, digital and paper-based flyers, and online information hubs accessible via the local intranet. The organization had not participated in the publication of an EMR online education program to a hospital-based learning platform previously. Each hospital site expressed the need for both digital teams and hospital-based education teams to endorse new online education materials.

All acute stroke teams worked within the context of rising caseloads and increasing number of stroke admissions per year, with resources and services under pressure. The observation of the 3 clinical contexts allowed the researchers to identify key considerations to be addressed within the education and implementation program (Table 1).

**Table 1:** Contextual considerations for implementation

Focus area	Contextual Considerations
Individual	Staff motivations and perceptions Individual digital literacy, staff availability and optimal timing for engagement Understanding disruptions or changes to individual clinical workflows
Team	Identification of teams involved (e.g., digital, education, clinical) Opportunities to talk about the technology (social learning/collective sensemaking) Team structure including staff turnover, team meetings Identification of key staff champions/leaders to facilitate ongoing learning Value of the intervention needs to be well communicated
Organization	Identification of governance structures: consider all levels Identification of health services leadership and support Provision of evidence-base for the technology Clear purpose identified and evaluation plans established

### Phase 3: theory

The assessment utilizing the NASSS-CAT revealed that 4 of the 7 domains were rated as complex encompassing the technology, the value proposition, the intended adopters, and the organization (see Additional File 2). This tool was used with the intention of developing strategies to target the most complex areas for implementation, while acknowledging that connections between the complexity domains and context would evolve simultaneously. The complexity domains are sequenced in accordance with the NASSS framework.

#### The technology

Complexity related to the stroke EMR enhancement included uncertainty about the feasibility of the end product replicating ideal design parameters. As the technology was the first of its kind within the organization, there were gaps in knowledge as to the technology’s performance (e.g., how will the data be extracted from the EMR to a national clinical registry). As a result, there were uncertainties about the technology’s usability and how to fit the technology efficiently within clinical workflows. The stroke EMR enhancement was also likely to require major changes to documentation practices regarding how clinical indicator data was collected, audited and shared.

#### The value proposition

The stroke EMR enhancement was designed to improve interprofessional collaboration via a single landing page offering clinicians enhanced visiblility and access to key clinical information, in turn supporting service quality and improvement. In addition, standardized data collection forms were designed to extract clinical indicator data for more efficient data extraction to a national clinical registry [38]. Key stakeholders recognized the value of the stroke EMR enhancement and were keen to pursue the implementation of the project and communicate this value proposition to frontline staff; this is illustrated by the quote below from a senior leader in the health service (Table 2).

**Table 2:** Key stakeholder perspective regarding value proposition of the stroke EMR enhancement

“And I can certainly see the substantial benefits it has…Just things like regular documentation of the NIHSS (National Institutes of Health Stroke Scale), so just for that monitoring of clinical improvement, ensuring that we've actually complied with all the acute care standards for acute stroke care….that it's cause for a thought process…And there's just so many safety and quality features, because I think some of it, at times, people felt, "Oh, you're just doing this as a data collection tool" – (but) that there's actually really strong safety and quality components that it actually facilitates, and ensures that we're compliant. And then that allows standardization of care. I know patient-centred care is very key, but in stroke, standardization of care, getting rid of that unwarranted variation is (also) actually really important, so that people aren't missing out on very pertinent steps in the care pathway”.	

Despite the percpetion of value to key stakeholders, the value of the stroke EMR enhancement to *frontline* staff was more uncertain as no EMR applications had been implemented at this scale previously within the organization. Additionally, the stroke EMR enhancement was a statewide change in practice. This standardization across multiple different hospitals and contexts provided uncertainty regarding the value proposition (e.g., some hospital contexts may have greater need for the intervention than others, or were there existing practices at certain hospitals that would compete with the benefit of the technology).

#### The intended adopters

The intended adopters of the stroke EMR enhancement were the clinical staff working in acute stroke wards across EMR sites in Queensland. Other staff that had the potential to be impacted were administation and data entry personnel, depending on the local procedures for clinical indicator data collection within hospitals. The complexity within this domain related to the uncertainty in how frontline staff would adopt the technology, with the possiblity of staff resistance to change and non-adoption if the value proposition was not well communicated. High staff turnover in acute stroke wards also poses a challenge to the adoption of the stroke EMR enhancement. Futhermore, acute stroke wards are busy and if staff did not feel they had the resources (e.g. time, space or support) to adopt changes to practice, uptake of the stroke EMR enhancement would be challenged.

#### The organization

The stroke EMR enhancement was one of the first clinically-led optimizations of the EMR driven by a statewide clinical network. The novelty of the intervention left organizational readiness for the innovation unclear, as the processes to embed them were not well established. This lack of established processes and guidelines for embedding the stroke EMR enhancement within the existing systems and workflows of the organization created an additional layer of complexity to the implementation process.

Key strategies for the education and implementation program design are presented in Table 3.

**Table 3:** The stroke EMR enhancement education and implementation program based on EPOC taxonomy: implementation strategies and explanatory activities/actions

Implementation Strategies	Activities/actions
*Targeting NASSS domain of technology*	
Provide educational materials	- Face-to-face group-based education sessions involving demonstration of use of the stroke EMR enhancement - Dissemination of QRGs and screenshots of profession specific details on how to use the technology - E-learning package
Conduct educational meetings	- Involvement in statewide stroke network meetings about the EMR enhancement rollout - Face-to-face training sessions scheduled as close to the rollout of the stroke EMR enhancement as possible (2–4 weeks)
Use reminders	- Reminder emails sent to staff champions to encourage staff engagement in education and training - QRGs used as posters on the ward to remind staff on how to use the technology
Involve local opinion leaders	- IT staff involved in the design and construction of the stroke EMR enhancement act as ‘go-to’ personnel for technical concerns
*Targeting NASSS domain of value proposition*	
Provide educational materials	- Communication of the purpose and value of the enhancement through the e-learning package - Dissemination of QRGs to guide new changes and convey purpose of the stroke enhancement
Conduct educational meetings	- Purpose and value of the stroke EMR enhancement communicated to staff in face-to-face group-based education sessions
Organize inter-professional education	- Staff encouraged to discuss the value of the enhancement to their own clinical practice (collective sensemaking)
Involve local opinion leaders	- Staff champions chosen to encourage engagement in education and training and promote the value of the intervention - E-learning package included quotes from key stakeholders and lead stroke physicians about the importance and value of the enhancement to demonstrate organizational support for the project
*Targeting NASSS domain of intended adopters*	
Provide educational materials	- E-learning package involved information on the clinician-led design of the EMR enhancement, including demonstration videos, quizzes and interactive lessons - QRGs provided easy ‘how-to’ lessons on use of the new features
Conduct educational meetings	- Face-to-face group training sessions designed to enhance interprofessional learning opportunities
Conduct educational outreach visits	- Face-to-face education and training sessions to enhance uptake at each hospital study site - Optimal timing for staff engagement in education sessions determined through site visits
Organize inter-professional education	- Clinicians encouraged to work with different professions in group-based training sessions and provide opportunities to share their learning experiences
Conduct local consensus processes	- Local workflows discussed with each staff champion to determine context appropriate implementation
Use reminders	- Posters, emails, TEAMS noticeboard and staff champions were used as reminders for staff to engage in the education and use of the stroke EMR enhancement
Involve local opinion leaders	- Staff champions encouraged engagement in education and training and optimal use of the enhancement
Provide tailored interventions	- Informal and formal feedback (interviews) of barriers to implementation and iterative changes to strategies for implementation where possible
*Targeting NASSS domain of the organization*	
Use reminders	- Communication to digital, clinical and education teams within health services about the enhancement and implementation

*EMR* Electronic Medical Record, *NASSS* Non-Adoption, Abandonment, Scale-Up, Spread and Sustainability framework, *QRG* Quick Reference Guide

### Phase 4: education and implementation program design and evaluation

Using knowledge from the literature, a blended approach to training was chosen for implementation of the stroke EMR enhancement. The blended approach involved e-learning modules, as well as face-to-face group-based training, and production and publication of Quick Reference Guides (QRGs), providing simple, condensed instructions on how to use the stroke EMR enhancement.

#### Group-based education

Face-to-face group-based education was delivered to medical, nursing and allied health staff through a series of brief, targeted sessions [46, 67] conducted by the researcher at each hospital site over a period of one to two weeks. To promote the uptake and utilization of the stroke EMR enhancement, designated staff champions were responsible for encouraging engagement amongst staff [44, 49, 65]. During group training sessions, a social aspect to the learning experience was encouraged [52] through discussions about the value and purpose of the EMR intervention for stroke care.

#### E-learning package

The interactive e-learning package included several modules:

1. Introduction: Background information on the EMR and overview of the stroke EMR enhancement.
2. Main features of the EMR enhancement: Three indivudual tutorials relating to features of the stroke EMR enhancement were presented to users.
3. How to use the enhancement: Video tutorials and interactive pages allowed users to understand use of the new features.
4. Quiz: A knowledge test included 2 multiple choice questions, 1 question involving matching correct answers and 1 written response.

**Fig. 2:**
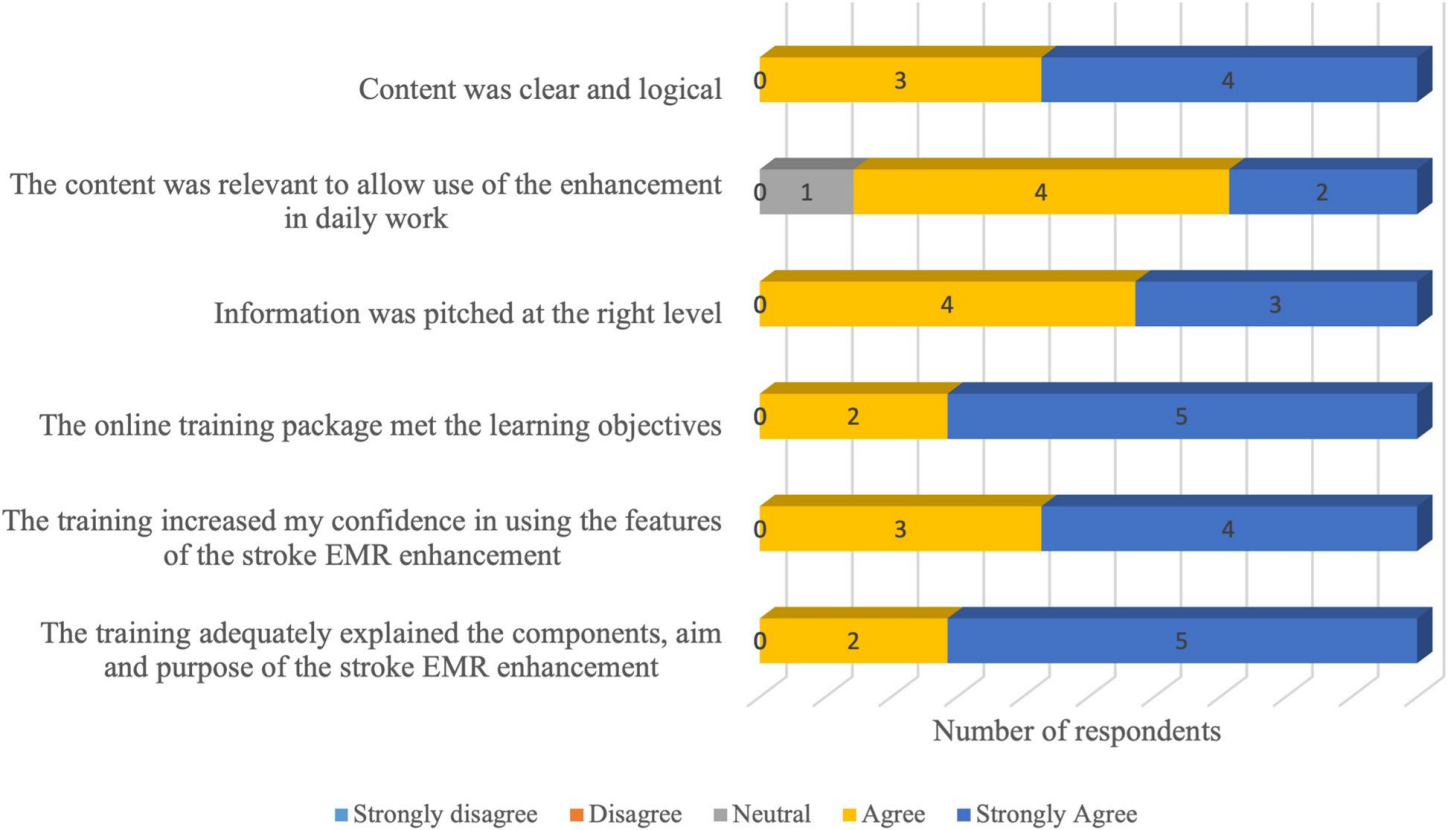
Statewide stroke network feedback for the e-learning package

The content and design of the e-learning was evaluted by seven members of the statewide stroke network (Fig. 2). Overall, the e-learning was rated as ‘very good’ (4/7) or ‘excellent’ (3/7) resulting in endorsement of the e-learning package for use within the education and implementation program.

The implementation and integration of the enhancement lasted three months at each site, during which time, key site personnel were encouraged to actively engage with the stroke EMR enhancement and promote the complementary e-learning package.

### Phase 5: implement and iterate

Data to inform the adjustments and iterations of the education and implementation program were gathered via staff feedback. Feedback was provided by clinical staff, site champions and nurse educators who were engaged with the education and implementation of the digital intervention. Researcher (SR) was involved in the education and implementation of the stroke EMR enhancement and in regular contact with the three health services and their staff champions, who acted as site principal investigators in the research. This regular communication provided the opportunity to gain informal feedback on barriers to engage staff in education and optimize processes for implementation at each study site. Barriers and challenges were also shared amongst site staff champions via meetings, emails and education sessions allowing collective sensemaking and adaptations to implementation. A fixed time-frame of three months of implementation and iterations was used at each study site. Where possible, all feedback was incorporated into further strategies for education and implementation. This process led to 4–8 iterations at study sites until no further changes were proposed within the implementation time-frame.

Action research made it possible to make iterative changes to implementation strategies based on feedback. For example, clinical staff indicated that the leadership and top-level support was not explicitly visible to ‘on-the-floor’ clinicians; increased visibility and communication of the EMR enhancement was required. The statewide stroke network was driving change through consultation with leading stroke clinicians at statewide and individual hospital and health service governance committees. Further top-down approaches were used to encourage uptake of the digital intervention, including presentations about the enhancement at key statewide network meetings, widespread email distribution of the project implementation and dissemination of QRGs to individual staff, health service digital health teams and acute stroke teams to support training and education.

Iterations were also made based on staff availability. Engaging clinical staff to complete training and education involved scheduling education sessions within existing clinical education time, e.g., Site 1 required small group (2–3 people) 15 min scrum sessions with computer demonstration whereas Sites 2 & 3 involved longer 40 min presentations with embedded computer demonstration. Education and training at Site 3 was interprofessional, with both nursing and allied health attending the same session compared to other sites where training took place separately between professions based on availability of clinicians. Table 4 presents the experienced barriers and strategies to overcome them [42].

**Table 4:** Iterative changes to implementation and education package based on barrier identification within contexts

Barrier	Study site	Strategy to address barrier	Adaptation to education and implementation program
*Targeting NASSS domain of the technology*			
Existing process for allied health documentation in the EMR meant that the EMR enhancement (mPage) was not populating necessary information	All sites	Tailored intervention	Profession specific QRGs were created and emailed to allied health staff members to facilitate change in documentation practice
Some hospitals only wanted to use certain elements of the EMR enhancement or quarantine use to particular members of the team to preserve data quality	Site 1 & Site 2	Tailored intervention	1:1 education sessions were performed with these key personnel and in some contexts, the focus of the education sessions was changed to suit the parts of the EMR enhancement supported by the team
*Targeting NASSS domain of value proposition*			
Digital health teams hesitant to deliver education and training on the ward as the project ownership came from the statewide stroke network and not health services specifically	Site 1 & Site 2	Tailored intervention	Digital health teams were made aware of the purpose and value of the stroke EMR enhancement through email communication and top-level support was offered to the project by key digital health leaders within health services
Clinical teams not recognising value or purpose of the project	All sites	Reminders	Staff were prompted to engage in education and training and staff champions were reminded about promoting the e-learning package
Value of parts of the technology was unclear to some key staff members due to existing processes for data collection and data extraction	Site 1 & Site 3	Educational outreach visit	Meetings with key staff members and the IT personnel who designed the stroke EMR enhancement were organized to discuss how the intervention could be of value within their context and how the technology could streamline processes for data collection and data extraction
*Targeting NASSS domain of intended adopters*			
Difficult to engage clinical staff often due to busy workloads, unstructured processes for education on ward and high turnover of clinical staff	All sites	Tailored intervention and reminders	The timing, length and approach to training and education was adjusted based on hospital context in order to capture as many staff as possible; some sites were offered small group (1–3) scrum style education sessions with computer demonstration whilst some involved interprofessional larger group workshops. Poster reminders were displayed on some wards
Fragmentation and lack of communication between teams (stroke/digital/education)	All sites	Educational meetings and materials	Each hospital acute stroke, digital and education team received communication about processes for distribution of education materials. QRGs were emailed to either digital health teams and/or CNCs (context specific) to encourage widespread dissemination of education materials
Uncertain uptake of e-learning package as staff need to access local learning platform to complete the online education	All sites	Reminders	Email to key staff members (CNC and Nurse educators) to ask to share availability of package in multidisciplinary team meetings and mention in face-to-face group education sessions
*Targeting NASSS domain of the organization*			
Organizational barrier of some health services being unaware of the project or unclear of the availability of the EMR enhancement in the system	All sites	Reminders	Statewide stroke network disseminated reminder email to members of the stroke network to encourage use of the EMR enhancement and availability of education materials. QRGs were disseminated to key personnel such as digital health teams and CNCs by the researcher
Individual hospital sites required their own governance processes for endorsing the education and training package despite statewide approval	All sites	Local opinion leaders	Top-down approach from statewide leadership to provide information via email on support of the project and endorsement of education materials

*EMR* Electronic Medical Record, *NASSS* Non-Adoption, Abandonment, Scale-Up, Spread and Sustainability framework, *QRG* Quick Reference Guide, *IT* Information Technology, *CNC* Clinical Nurse Consultant

Overall, the NASSS framework supported the ability to identify barriers and suggest optimal strategies to target complexity domains. As expected, barriers were identified across all domains of the framework deemed complex.

## Discussion

Implementation of digital interventions in healthcare settings is complex, especially when they require integration into existing workflows and processes [73]. The lack of research on how to develop effective education and implementation programs for digital interventions in clinical settings hinders efforts to improve digital health implementation [13, 32]. A pragmatic evidenced-based and theory-driven education and implementation program was developed for implementation of the stroke EMR enhancement. Although the literature clearly states the use of theory and evidence are essential to develop and evaluate implementation of interventions [74–77], few studies report on the procedural details of such education and implementation strategies [31]. Guidance from the UK Medical Research Council for developing and evaluating complex interventions states that “alongside implementation-specific outcomes (such as reach or uptake of services), attention to the components of the implementation strategy, and contextual factors that support or hinder the achievement of impacts, are key” [75]. This study addressed these recommendations by using the NASSS framework combined with evidenced-based education and training strategies to develop targeted approaches for mitigating implementation complexity, while also documenting adaptations to the implementation process.

### The NASSS framework to guide education and implementation

Our study contributes to the growing literature on usefulness of the NASSS framework for implementing and evaluating digital interventions in healthcare [34, 78, 79]. The NASSS framework was useful in identifying complex domains within implementation and enabled the development of targeted education strategies for multiple users and contexts. For instance, education strategies to enhance the technical skills of end-users, through face-to-face and hands-on training, targeted the technology domain. Additionally, strategies such as engaging key stakeholders and local opinion leaders aimed to manage organizational complexity by enhancing coordination and communication. The domains of the value proposition and intended adopters were considered to be most modifiable through education and training.

The NASSS framework identifies the value proposition as a critical factor in technology implementation, which is also emphasized in other implementation studies [33, 80]. The value proposition encompasses not only the perspectives of end users and adopters, but also those of key stakeholders involved in the technology design and implementation. Different stakeholders can hold different views of the value of a technology. Given the range of statewide and local stakeholders in this study, it was important to ensure the value of the stroke EMR enhancement was transparent at all levels and aligned with organizational objectives. For instance, local opinion leaders in the form of staff champions were employed to promote the value proposition, and the e-learning package included compelling quotes from statewide stroke physicians about the enhancement's value for clinical practice. It was anticipated that a clear comprehension of the value proposition would facilitate the incorporation of the enhancement into routine clinical practice.

The intended adopters of the stroke EMR enhancement were clinical staff comprising medical, nursing, and allied health professions. The effectiveness of the intervention in streamlining data visibility and data collection depended on the extent of staff engagement. Non-adoption of the technology by staff posed a risk to the system's ability to achieve its intended goals. It was observed that planned education strategies, such as face-to-face and interprofessional group-based sessions, required tailoring and customization to align with contextual demands. This highlights the need for flexibility and adaptability in the implementation of digital health technologies. The NASSS framework was feasible to implement and provided a practical guide in the prospective planning of education and implementation strategies, enabling the researchers to reflect on the project's evolving complexities [34].

### Context, adaptations and complexity

The stroke EMR enhancement is a standardized technical solution aiming to enhance data visibility, documentation and data extraction within the EMR. Despite implementing a standardized technical solution, implementation plans are unlikely to succeed if they are standardized across different contexts [8–10]. Our study findings align with what is highlighted in the literature – context is a central component within implementation [8]. In this case, barriers faced were sometimes universal, although most often context specific, such as the composition of multidisciplinary teams, the adoption of some aspects of the intervention and not others, and the lack of engagement of some key staff members. According to complexity science, it is difficult to anticipate these obstacles before implementation and they must instead be overcome as the implementation process unfolds [3, 16].

The iterative approach we adopted in the design and execution of the education and implementation program allowed for context specific iterations to promote optimal implementation at each study site. In line with previous literature [31, 81], planning for iterations within the implementation process can allow for better ‘innovation fit’ to the context. The education and implementation program design allowed for customization of strategies to fit the hospital contexts (e.g., approaches to training, timing of training and dissemination strategies), aligning with individual hospital processes and cultures.

Our research study aimed to address the substantial gap in the availability of evidenced-based educational programs for implementation of complex digital interventions [7]. Recognizing the intricacies of education and training in this complex domain has been described as a 'wicked problem' [22]. Rangel et al. [22] call for “new theoretical lenses” to incorporate complexity within EMR training approaches, departing from the conventional linear causal relationship analysis. Aligned with this concept, our research is grounded in complexity theory [82], utilising a multi-phased approach to combine the NASSS framework with evidence-based education and training strategies for implementation. The phases of development recognise complexity, context and the necessary iterations and adaptations required during development of the education and implementation program. While education and training continue to play a crucial role in implementing complex digital interventions in healthcare, further research is needed to understand how technology advancements and user adoption may influence the role and essential components of education and training in this evolving landscape.

### Study limitations and strengths

Several limitations are evident within this research study. The research team had initially planned to study implementation of the stroke EMR enhancement at an initial site only, and then co-design education and training strategies with staff from this initial site through interview and focus group feedback. Due to the delay in the production of the stroke EMR enhancement induced by the Covid-19 pandemic, this co-design aspect of the study was not able to be executed. Nevertheless, the findings of this study demonstrated the need for co-designing the implementation process with multiple sites due to the contextual variability and complexity across sites, highlighting the potential limitations of the original study plan. Additionally, despite governance and leadership from the statewide stroke network, the researcher acted as the educator and project manager during implementation. This positionality may have skewed barrier and enabler identification and may affect sustainability of the intervention once the research is complete. Furthermore, measuring behaviour change and outcomes after iterations to the education was outside of the scope of this study. Measuring the effectiveness of the education and implementation will be presented in further research. Strengths of this study include the use of a theoretical and evidence-based approach to the development of the education and implementation program. This approach allowed for the identification and prioritization of education and implementation strategies that were most likely to effectively address the high complexity elements of the implementation process. This paper makes a valuable contribution by providing a comprehensive account of the education and implementation program components, and contextual factors that may influence its uptake – this is a complex process that requires a multi-phased approach. The suggested approach could serve as a framework for clinicians, managers, and educators in developing education and implementation programs for complex digital interventions.

## Conclusion

Education is a critical component within the implementation of complex digital healthcare interventions. To implement the stroke EMR enhancement, a multi-phased approach was employed, incorporating the NASSS framework, evidence-based education and training strategies, and context assessments. The approach emphasized the documentation of adaptations made to the education and implementation program to address complexity and context. The NASSS framework proved useful when used as a guide to prospectively develop an education and implementation program, acknowledging complexity of the digital intervention. The effectiveness of the program in enhancing the adoption and utilization of the stroke EMR enhancement will be evaluated in future mixed methods research.

### Supplementary Information

**Additional file 1.** The digital intervention: stroke EMR enhancement.

**Additional file 2.** Complexities in the stroke EMR project assessed by the NASSS-CAT.

## Data Availability

The datasets used and/or analysed during the current study are available from the corresponding author on reasonable request.

## Abbreviations

EMR: Electronic Medical Record
CAS: Complex Adaptive System
NASSS: Non-adoption, Abandonment, Scale-up, Spread and Sustainability
NASSS-CAT: Non-adoption, Abandonment, Scale-up, Spread and Sustainability Complexity Assessment Toolkit
IT: Information Technology
CNC: Clinical Nurse Consultant
EPOC: Effective Practice and Organization of Care
QRG: Quick Reference Guide
