# Supplementary material for: Addressing complexity when developing an education program for the implementation of a stroke Electronic Medical Record (EMR) enhancement
Source: BMC Health Serv Res. 2023 Nov 24;23:1301. doi: 10.1186/s12913-023-10314-z (PMC10675965; doi:10.1186/s12913-023-10314-z)
Supplement: Supplementary file 1 — Additional file 1. The digital intervention: stroke EMR enhancement. [file 12913_2023_10314_MOESM1_ESM.docx]

**Additional File 1. The digital intervention: stroke EMR enhancement**

| **Characteristics of the stroke EMR enhancement** | |
| --- | --- |
| Geographical setting | Queensland EMR sites – case study involves evaluation at 4 hospital sites |
| Target population and condition | Clinical staff working in the acute stroke unit at each hospital site |
| Technology | An electronic medical record (EMR) enhancement tool that:   1. incorporates an EMR landing page display for visualisation of key clinical information offering a visual overview of progress and care planning 2. incorporates four standardised data collection forms (tick/flick boxes) for documentation and data collection, enabling more efficient data extraction processes 3. extracts data from the enhancement for upload to a national clinical registry |
| Sector | Secondary and tertiary care  Public health service |
| Main goal of project | Improving interprofessional practice, efficiency of health service delivery and digitisation of data input to the national clinical registry |
| Policy context | Rising caseload/ High numbers of acute stroke patients, limited staff resources and services under pressure |
| Project framed primarily as | Improving efficiency and enabling use of digital enabled technologies |

| **Components of the stroke EMR enhancement** | |
| --- | --- |
| Stroke summary page | 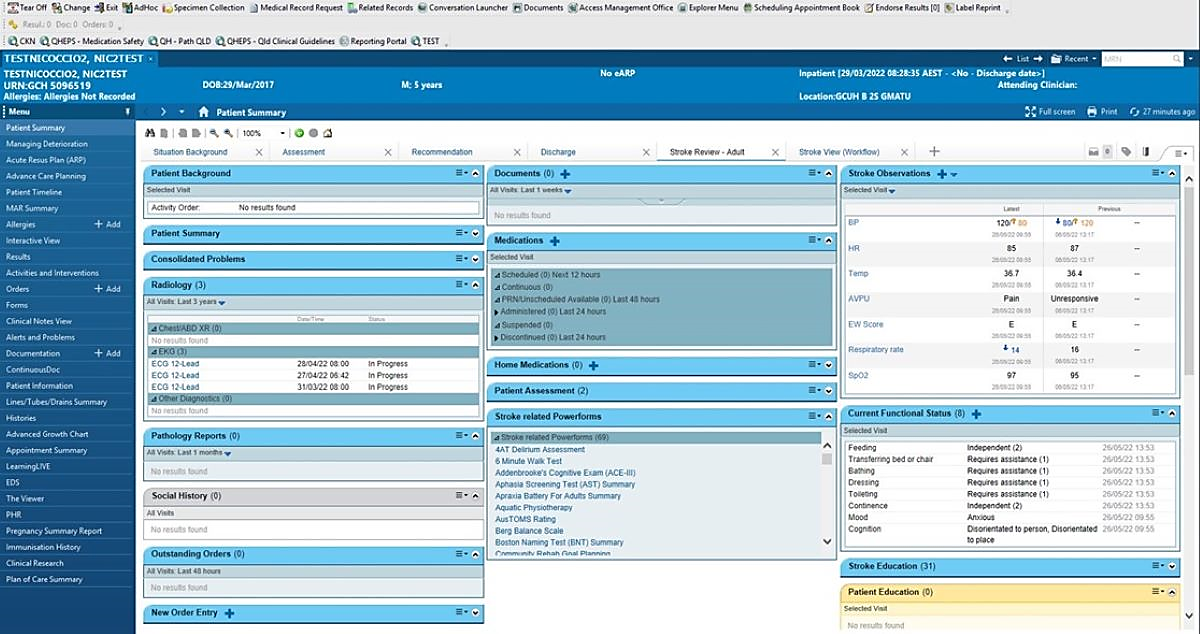  A single landing page offering a consolidated view of a patient’s key clinical information, to enhance information visibility and interprofessional collaboration.  The stroke summary page includes new clinical features (highlighted) added to the usual summary page view including links to radiology results, pathology results, stroke related data collection forms, current functional status, stroke education and National Institutes of Health Stroke Scale (NIHSS) score. |
| Data collection forms | 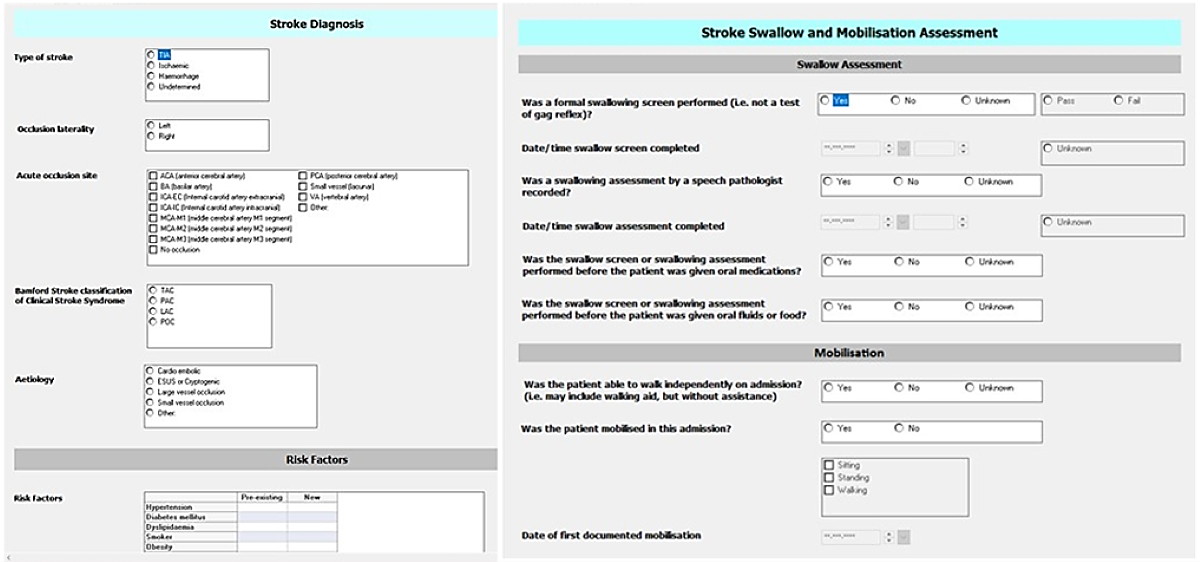Example of two of the four data collection form templates demonstrating clinical indicator data requirements and simple tick boxes for documentation.  The four data collection form templates are: 1) Stroke Initial Assessment; 2) Stroke Diagnosis & Interventions; 3) Stroke Swallow and Mobilisation Assessment; 4) Stroke Discharge Care |
| Data extraction tool | Information from the data collection forms directly aligns with national clinical indicator registry data. The EMR provides a tool to extract the information from these data collection forms into a comprehensive excel spreadsheet for efficient upload to the online data management portal. |
