# Supplementary material for: Addressing complexity when developing an education program for the implementation of a stroke Electronic Medical Record (EMR) enhancement
Source: BMC Health Serv Res. 2023 Nov 24;23:1301. doi: 10.1186/s12913-023-10314-z (PMC10675965; doi:10.1186/s12913-023-10314-z)
Supplement: Supplementary file 2 — Additional file 2. Complexities in the stroke EMR project assessed by the NASSS-CAT. [file 12913_2023_10314_MOESM2_ESM.docx]

**Additional File 2. Complexities in the stroke EMR project assessed by the NASSS-CAT**

| **NASSS Domain** | **Agree** | **Disagree** | **Not applicable/Don’t know** | |
| --- | --- | --- | --- | --- |
| **The illness or condition** | The condition is acute stroke which is a highly multidisciplinary area of clinical care involving assessment and diagnoses from often multiple health professionals e.g. doctors, social work, dietitian, physiotherapy, occupational therapy, speech pathology. For optimal rehabilitation, stroke teams are required to work in interprofessional teams with both clinicians, patients and families. Stroke care involves multiple areas of standardized clinical care and monitoring in order to achieve optimal patient outcomes. | | | |
|  | **Identifying complexity** | | | **Stroke EMR enhancement** |
|  | There are significant uncertainties about the illness or condition | | |  |
|  | Many people with the condition have other co-existing illnesses or impairments that could affect their ability to benefit from the technology or service | | |  |
|  | Many people with the condition have social or cultural factors that could affect their ability to benefit from the technology or service. | | |  |
|  | | | | |
| **The technology** | The technology is the stroke EMR enhancement which involves 3 core components: 1) a single landing page (mPage) which is an one page visualisation of nursing, medical and allied health clinical information 2) documentation and data collection forms (powerforms) which assist in standardised data collection and 3) extraction of clinical indicator data for a national clinical stroke registry.  This is different to the current EMR in which information is siloed into different page views which can involve multiple clicks into and out of the electronic notes, cognitive burden and difficulty in visualising information. Also, the current EMR system does not allow data extraction of the routine clinical information documentation. | | | |
|  | **Identifying complexity** | | | **Stroke EMR enhancement** |
|  | There are significant uncertainties about what the technology is | | |  |
|  | There are significant uncertainties about where the technology will come from | | |  |
|  | There are significant uncertainties about the technology’s performance and dependability | | |  |
|  | There are significant uncertainties about the technology’s usability and acceptability | | |  |
|  | There are significant technical interdependencies | | |  |
|  | The technology is likely to require major changes to organisational tasks and routines | | |  |
|  | | | | |
| **The value proposition** | The technology is designed to improve interprofessional collaboration, communication and coordination via a single landing page allowing improved visibility of clinical information and workload efficiency. Also, the premise of the stroke EMR enhancement is to be able to extract clinical indicator data that clinicians routinely document for clinical care. Currently, staff are required to enter data manually into the national clinical stroke registry database; the stroke EMR enhancement will leverage the EMR to improve this process and enable increased efficiency of data extraction. | | | |
|  | **Identifying complexity** | | | **Stroke EMR enhancement** |
|  | The commercial value of the technology is uncertain | | |  |
|  | The value to the patient or client is uncertain | | |  |
|  | The value to the clinician or other staff member is uncertain | | |  |
|  | The value to the healthcare system is uncertain | | |  |
|  | The value to this particular healthcare organisation is uncertain | | |  |
|  | The technology could generate a negative value (i.e., costs are likely to outweigh benefits) for some stakeholders. | | |  |
|  | | | | |
| **The intended adopters** | The intended adopters of the technology are the professional clinical staff in acute stroke areas across EMR sites in Queensland. Other staff that may be impacted include admin staff, which may be involved in the data extraction procedure depending on each health service local procedures. | | | |
|  | **Identifying complexity** | | | **Stroke EMR enhancement** |
|  | There is uncertainty about whether and how patients/carers or citizens will adopt the technology | | |  |
|  | There is uncertainty about whether and how front-line staff will adopt the technology | | |  |
|  | There is uncertainty about the implications for people who might be indirectly affected by the technology | | |  |
|  | | | | |
| **The organisation** | The organisation is a public health service in Queensland with an integrated single-instance EMR. Stakeholders include Queensland hospital sites and eHealth departments. The statewide stroke network provided leadership for the design of the enhancement. As the enhancement was clinically-led in its design, a number of key clinical stakeholders for each profession were involved in the development and iterations of the design of the enhancement. The stroke EMR enhancement is the first of its kind in the organisation to implement a digital health technology statewide therefore there is no historical track record to work from. There was initial investment in the creation of the stroke EMR enhancement in terms of clinician time, a project officer and a small financial outlay from the organisation although funding did not progress across the lifespan of the project. | | | |
|  | **Identifying complexity** | | | **Stroke EMR enhancement** |
|  | The organisation’s capacity to take on technological innovations is limited | | |  |
|  | The organisation is not ready for this particular innovation | | |  |
|  | Organisational routines and processes will need to change very considerably to accommodate the technology | | |  |
|  | Procurement processes are in place that make it harder to commission this technology | | |  |
|  | The work needed to introduce and routinise the innovation has been underestimated and/or inadequately resourced | | |  |
|  | | | | |
| **The external context** | There are national guidelines for the management of acute stroke patients and a national clinical registry that assesses adherence to guidelines, facilitating ongoing service and quality improvement. The statewide stroke network acts as a professional body within Queensland to enhance the provision of evidenced based stroke care and develop improvement activities based around quality data collection. | | | |
|  | **Identifying complexity** | | | **Stroke EMR enhancement** |
|  | The political and/or policy climate is adverse | | |  |
|  | Professional organisations are opposed to the innovation or don’t actively support it | | |  |
|  | Patient organisations and lobbying groups are opposed to the innovation or don’t actively support it | | |  |
|  | The regulatory context is adverse | | |  |
|  | The commercial context is adverse | | |  |
|  | Opportunities for learning from other (similar) organisations are limited | | |  |
|  | Introduction of the technology/innovation could be threatened by external changes that impact on the organisation | | |  |
|  | | | | |
| **Emergence over time** | There is some uncertainty around the EMR technology in the future; currently Cerner is the EMR vendor and if this changes in the future, then the technology will need to be adapted to a new server. Also, the data extraction technology of the enhancement could be replaced with another technology which is able to do the same task. There could be a change in the value proposition or individual perception of the technology which could be both positive or negative. The most significant key uncertainty is probably around the adoption of the technology by staff and value proposition: there is uncertainty around the perception of the enhancement, use of the enhancement and success of the enhancement to meet its goals underpinned by whether the staff use the enhancement and think it is valuable. | | | |
|  | **Identifying complexity** | | | **Stroke EMR enhancement** |
|  | The population with the condition, and/or how the condition is treated, is likely to change significantly over the next 3-5 years | | |  |
|  | The technology (and/or the service model it supports) is likely to change significantly over the next 3-5 years | | |  |
|  | The value proposition for the technology is likely to change significantly over the next 3-5 years | | |  |
|  | There will be significant changes to individual users’ perceptions of the technology over the next 3-5 years | | |  |
|  | The organisation(s) involved are likely to have significant restructurings or changes in leadership, mission or strategy over the next 3-5 years. | | |  |
|  | The policy, regulatory and economic context for this innovation is likely to be turbulent over the next 3-5 years | | |  |
